# Evaluation of the biodiversity of arbuscular mycorrhizal fungi during regenerative succession in quarries

**DOI:** 10.18699/vjgb-25-09

**Published:** 2025-02

**Authors:** A.A. Kryukov, A.P. Yurkov, A.O. Gorbunova, T.R. Kudriashova, A.I. Gorenkova, Y.V. Kosulnikov, Y.V. Laktionov

**Affiliations:** The All-Russia Research Institute for Agricultural Microbiology, Pushkin, St. Petersburg, Russia; The All-Russia Research Institute for Agricultural Microbiology, Pushkin, St. Petersburg, Russia; The All-Russia Research Institute for Agricultural Microbiology, Pushkin, St. Petersburg, Russia; The All-Russia Research Institute for Agricultural Microbiology, Pushkin, St. Petersburg, Russia; The All-Russia Research Institute for Agricultural Microbiology, Pushkin, St. Petersburg, Russia; The All-Russia Research Institute for Agricultural Microbiology, Pushkin, St. Petersburg, Russia; The All-Russia Research Institute for Agricultural Microbiology, Pushkin, St. Petersburg, Russia

**Keywords:** arbuscular mycorrhizal fungi, biodiversity, regenerative succession, quarry, Illumina, грибы арбускулярной микоризы, биоразнообразие, восстановительная сукцессия, песчаный карьер, Illumina

## Abstract

Arbuscular mycorrhizal fungi (AMF) play a key role in the regenerative successions of plant communities after anthropogenic disturbances, particularly in quarries. AMF help plants with water and mineral nutrition, contributing to the restoration rate of vegetation cover. The research is aimed to study the biodiversity of AMF using molecular genetic methods at different stages of overgrowth of two quarries in the Leningrad region. Molecular genetic identification of fungi was carried out using Illumina MiSeq analysis of the ITS1 and ITS2 regions as barcodes for the identification of operational taxonomic units (OTUs) with species-level identification. An adapted and error-checked AMF genetic sequence database from NCBI was used as a reference. The study applied an optimized nucleic acid isolation technique for sandy soils. The results showed maximum AMF biodiversity at the initial stages of overgrowth – pioneer and grass stages – with minimum diversity observed at the shrub stage, where it decreased by five times. At the forest stage, the biodiversity of AMF was almost restored to the level seen at the grass stage. It has been shown that the biodiversity and species composition of AMF can vary greatly between the stages of regenerative succession and probably depends primarily on the biodiversity of grasses, with which AMF most effectively enter into symbiotic relationships. The analysis showed a reliable negative correlation between the number of AMF species and the number of woody plant species. Such studies can aid in understanding how plant-fungal symbiosis develops in regenerative successions and which AMF most effectively contribute to vegetation cover restoration

## Abstract (ru)

Грибы арбускулярной микоризы (АМГ) играют ключевую роль в восстановительных сукцессиях растительных сообществ после антропогенных нарушений, в частности на песчаных карьерах. АМГ помогают растениям в водном и минеральном питании, ускоряя восстановление растительного покрова. Целью исследования было изучить биоразнообразие АМГ молекулярно-генетическими методами на разных стадиях зарастания двух карьеров Ленинградской области. Молекулярно-генетическая идентификация грибов проводилась с использованием анализа Illumina MiSeq по регионам ITS1 и ITS2 в качестве баркода для поиска операционных таксономических единиц с идентификацией микроорганизмов до вида. Референсом служила адаптированная и проверенная на ошибки база генетических последовательностей АМГ из NCBI. В исследовании использовалась оптимизированная для песчаных почв методика выделения нуклеиновых кислот. Максимальное биоразнообразие АМГ наблюдалось на начальных стадиях зарастания – пионерной и злаковой. На кустарниковой стадии разнообразие снижалось в пять раз, а затем на лесной стадии восстанавливалось почти до уровня злаковой. Биоразнообразие и видовой состав АМГ могут значительно меняться между стадиями восстановительной сукцессии, что может в первую очередь зависеть от биоразнообразия трав, с которым АМГ вступают в симбиотические отношения наиболее эффективно. Показана достоверная отрицательная корреляция между числом видов АМГ и числом видов древесных растений. Проведенное исследование может помочь в понимании того, как развивается растительно-грибной симбиоз в восстановительных сукцессиях и какие АМГ наиболее эффективно помогают в восстановлении растительного покрова.

## Introduction

Most Embryophytes (more than 90 % of the families) form arbuscular mycorrhiza with fungi (AMF) of the Glomeromycotina subdivision, the Mucoromycota division (Spatafora et al., 2016). AMF help the symbiotic plant with water and mineral nutrition, receiving complex organic substances in return. In regenerative successions, mycorrhizal interaction promotes the plant’s ability to compete and overcome unfavorable edaphic conditions (van der Heijden et al., 1998; Lambers et al., 2008). Mycorrhiza presence may be an influential factor contributing to the successful absorption of free substrates by plants. Mycorrhizal symbionts can significantly enhance the growth conditions of pioneer plants. Mycorrhiza is a crucial adaptation for plants facing deficiencies in nitrogen, phosphorus, and other essential minerals, unfavorable water, air conditions, and a lack of organic carbon substrates (Aikio, 2000). AMF is likely to facilitate succession, but as of now, there is very little direct evidence of this in natural ecosystems (Smith, Read, 2008). The species composition of AMF communities is known to have a great influence on plant productivity, plant community structure, successional patterns, and ecosystem performance (Wu, 2017). The quantitative and species composition of micromycetes is mainly determined by the vegetation (Sumina et al., 2010). The diversity and performance of mycorrhizal fungi are crucial for biodiversity and ecosystem health, while the diversity and structure of vegetation can also influence the diversity of AMF populations (Jeffries, Barea, 2001). AMF diversity is expected to be lower in the quarry substrate than in zonal soils, as there is lower soil moisture content, which is fundamental to the very survival of these microorganisms (Ganugi et al., 2019).

Soil microbial biodiversity analysis uses high-throughput sequencing methods, primarily with Illumina MiSeq. Various genetic markers are used to identify AMF, generally by using an ITS (internal transcribed spacer) region or the SSU (Small SubUnit ribosomal gene fragments) and LSU (Large SubUnit) gene regions that flank it (Kryukov et al., 2020). In some cases, it involves other genes or even full-genome sequencing. The lack of consensus on a barcode marker in Glomeromycotina provides challenges to ecological and phylogenetic studies. The conserved regions of the SSU and LSU genes are suitable for effective AMF identification to the level of genus, however, they are poorly fit for species-level identification due to their low variability (Öpik et al., 2014). To effectively perform an AMF species identification, it is preferable to use variable ITSs, although it can often lead to the identification of virtual taxa (Kryukov et al., 2020).

This study aims to assess the AMF biodiversity using molecular genetic methods at different overgrowth stages of two quarries in the Leningrad region, Russian Federation

## Materials and methods

The materials were collected in midsummer of 2018 and 2019 at two quarries of different ages in the Vsevolozhsk district of the Leningrad region: Kuzmolovo (60.116448N, 30.545006E) and Kalelovo (60.256590N, 29.971972E). Pioneer stage communities at the quarries were sparsely closed, with total projective cover by plants (TPC) not exceeding 20 %. During the grass stage, Gramineae dominated the communities, which were predominantly composed of various grasses. During the shrub stage, the upper tier of the communities was composed of shrubs and undergrowth. The forest stage was composed of young growths of the forest vegetation type. Sample plots measuring 5 × 5 meters were established at each stage of the regenerative succession in the quarries (Gorbunova, Sumina, 2021). To conduct the genetic AMF composition analysis, 20 plots were sampled, four sites per succession stage. Soil sampling was conducted in the rhizosphere of each plot. For five plant species (Agrostis capillaris, Artemisia vulgaris, Chamaenerion angustifolium, Deschampsia cespitosa, and Tussilago farfara), up to 25 soil samples were collected per plot, with five samples taken for each species whenever possible. According to the published works (Wang, Qiu, 2006; Akhmetzhanova et al., 2012), these plant species form symbiosis with AMF. Botanical description of the plots and plant mycorrhization level assessment were performed earlier (Gorbunova, Sumina, 2021).

An optimized technique involving Illumina MiSeq sequencing was used for AMF molecular genetic identification (Kryukov et al., 2020; Yurkov et al., 2024). Rhizosphere soil samples containing AMF mycelium and spores were taken for identification. For DNA extraction, a 0.5 g sample of frozen soil and 1 g of garnet abrasive were taken into a 2 mL tube for mechanical grinding (Pinaev et al., 2022). After that, 700 μL of CTAB buffer (2 % CTAB; 1.4 M NaCl; 20 mM EDTA; 100 mM Tris-HCl pH = 8.0) was added to the heated tube containing soil. The tubes were shaken in a vortex mixer for 1 minute every 15 min and incubated at +65 °C for up to 2 h. After thermal, chemical, and mechanical treatment, the samples were centrifuged for 5 min, and then the supernatant was transferred to new test tubes. A second DNA washing was conducted using 500 μL of water. This process involved shaking the soil and water mixture for 5 minutes. After centrifugation, the second supernatant was combined with the first supernatant. Additional washing with water was imperative as DNA tends to adsorb on sandy soil particles. The obtained DNA was freed from impurities by double extraction with an equal volume of chloroform. After each centrifugation (10 min at 14,000 rpm, Eppendorf, Germany), the supernatant with DNA was sampled and transferred to a new tube. The DNA was precipitated with 2/3 V isopropanol with 0.4 M NaCl, washed with 70 % ethyl alcohol, dried for 3 min, and then further dissolved in water (Maniatis et al., 1982). The DNA was purified with the AMРure XP magnetic particles (Beckman Coulter, USA).

The purified DNA was used for a separate PCR of the ITS1 and ITS2 marker regions with universal primers. The primers were synthesized in Evrogen (Russia) with the 5′-TCG TCGGCAGCGTCAGATGTGTATAAGAGACAG-3′ adaptor for forward primers and the 5′-GTCTCGTGGGCTCGG AGATGTGTATAAGAGACAG-3′ adaptor for reverse pri- mers for Illumina MiSeq: ITS5 (5′-GGAAGTAAAAGTCG TAACAACAAGG-3′) and our modified ITS-2RK reverse primer (5′-CGTTCAAAGATTCGATGATTCAC-3′) for the ITS1 amplification; the ITS3 primers (5′-GCATCGAT GAAGAACGCAGC-3′) and the ITS4 reverse primer (5′-TC CTCCGCTTATTGATATGC-3′) for the ITS2 amplification. After the first PCR round with 20 cycles, the PCR product was diluted 100 times and reamplified with 30 cycles. After amplification and visualization on an agarose gel, the PCR products of ITS1 and ITS2 were pooled for each sample and purified using AMPure XP magnetic particles (Beckman Coulter, USA).

Prior to sequencing, the purified amplicon libraries from each plot were combined to create a single sequencing run that reflects the AMF species composition for each plot. The amplicon libraries were sequenced on Illumina MiSeq using the MiSeq® Reagent Kit v3 (600-cycle) with paired-end reads (2 × 300 n.) (Illumina, Inc., USA). The identified sequences were then processed with the Illumina software (Illumina, Inc., USA). Illumina MiSeq sequencing resulted in FASTQ sequences from forward and reverse primers. This format covers sequence data and quality scores for each nucleotide position. The sequencing results have been submitted to the NCBI database (https://www.ncbi.nlm.nih.gov/bioproject/ PRJNA997898/, BioProject ID: PRJNA997898).

The methodology for the bioinformatics analysis is detailed in our 2020 research (Kryukov et al., 2020). In the present study, the data analysis was performed using the local database of reference sequences. As a starting point, we took the sequences from the NCBI database and filtered them for errors. Authorship, sequencing year, manual alignment analysis, and phylogenetic analysis were reviewed to filter any errors. Currently, our database contains data on 33 genera and 176 AMF species.

## Results

To analyze the AMF biodiversity in four stages of regenerative succession (Table 1), 20 sequencing runs were performed with a depth of up to 100,000 reads per sample (4 sequencing runs for each succession stage for the two quarries). Bioinformatic data processing was performed with USEARCH software (Edgar, 2010). We also calculated the Margalef and Shannon diversity indexes, as well as the Williams polydominance index (Table 1).

**Table 1.**
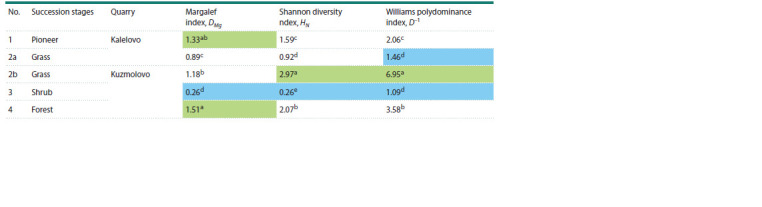
AMF diversity and polydominance indexes at plots of different succession stages Note. The “a–d” indexes indicate significantly different values of the estimated parameter (p < 0.05). Green indicates higher values; blue indicates lower values

We searched for the fungi OTUs (Operational Taxonomic Units) of different divisions (Table 2) for four succession stages in the two quarries. OTUs can denote both real individual species and virtual taxa (having no close reference in the database) of different taxonomic levels

**Table 2.**
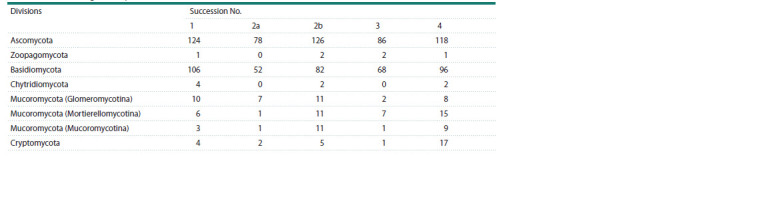
Identified fungi OTUs by division

The lowest number of the AMF OTUs (Mucoromycota (Glomeromycotina)) has been found at the shrub stage, and the highest, at the grass stage in Kuzmolovo. Interestingly, this is different for other fungal divisions; for example, the highest biodiversity of Ascomycota and Basidiomycota fungi was observed at the pioneer stage of the regenerative succession.

**Fig. 1.**
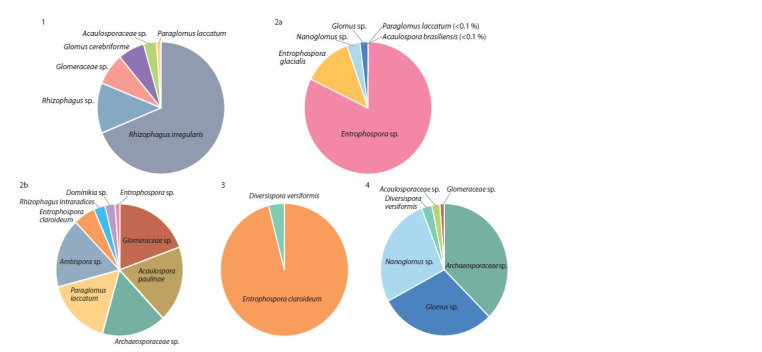
Glomeromycotina species composition at different stages of regenerative succession (proportion of reads). Succession numbers correspond to Table 1.

About half of the identified AMF OTUs were able to be positively assigned to species (Fig. 1). Figure 1 shows both the identified species and the read count proportion during sequencing (in percentages), which can suggest their occurrence at each succession stage. 1 (pioneer stage, Kalelovo) – Rhizophagus irregularis (68.9 %), Rh. sp. (12.2 %), Glomeraceae sp. (7.8 %), Glomus cerebriforme (6.7 %), Acaulosporaceae sp. (3.3 %), Paraglomus laccatum (1.1 %); 2a (grass stage, Kalelovo) – Entrophospora sp. (81.2 %), E. glacialis (12.6 %), Nanoglomus sp. (3.5 %), Glomus sp. (2.1 %), Paraglomus laccatum (0.1 %), Acaulospora brasiliensis (0.1 %); 2b (grass stage, Kuzmolovo) – Glomeraceae sp. (19.8 %), Acaulospora paulinae (18.0 %), Archaeosporaceae sp. (17.1 %), Paraglomus laccatum (16.1 %), Ambispora sp. (16.0 %), Entrophospora claroideum (5.6 %), Rhizophagus intraradices (3.2 %), Dominikia sp. (2.8 %), Entrophospora sp. (1.4 %); 3 (shrub stage, Kuzmolovo) – Entrophospora claroideum (95.6 %), Diversispora versiformis (4.4 %); 4 (forest stage, Kuzmolovo) – Archaeosporaceae sp. (37.7 %), Glomus sp. (29.3 %), Nanoglomus sp. (27.2 %), Diversispora versiformis (2.6 %); Acaulosporaceae sp. (2.1 %), Glomeraceae sp. (1.1 %). Interestingly, R. irregularis is most prevalent at the pioneer stage; it is replaced by other AMF later on.

**Fig. 2.**
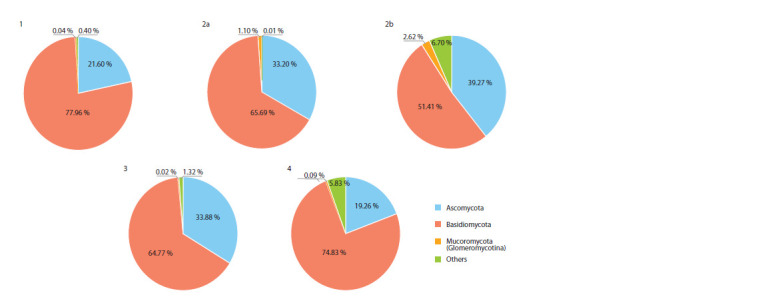
Read proportion based on the Illumina MiSeq sequencing results for the fungal kingdom divisions. Succession numbers correspond to Table 1.

Figure 2 shows the proportion of read counts after Illumina MiSeq sequencing for major fungal divisions. The Basidiomycota division fungi are most represented at all succession stages. Basidiomycota reads are most abundant during the pioneer stage; their proportion decreases at the grass stage, increases again at the shrub stage, and most significantly, at the forest stage. The share of Ascomycota fungi is also significant. The fungi of other divisions are scarcely represented in the DNA array at all stages of regenerative succession. At the grass stage, it is important to note that AMF species are represented by the highest number, and they also exhibit the greatest total proportion of reads compared to other stages. In general, the proportion of AMF reads is not high if compared to other fungal divisions; this has been found previously in other studies. The maximum read percentage for AMF by ITS using universal primers amounts to up to 2 % (Senés-Guerrero, Schüßler, 2015).

Correlations were analyzed between AMF species, species from other fungal divisions, and various parameters taken from the study by A.O. Gorbunova and O.I. Sumina (2021). These parameters included the number of grass species, moss species, woody plant species, total projective cover of plants, and projective cover by plant group. The calculations were performed across different stages of regenerative succession. A strong negative correlation was found between the number of AMF species and the number of woody plant species (–0.85).

## Discussion

The results are generally consistent with the global trends observed in AMF research. The highest AMF biodiversity in our work is found at the grass stage of regenerative succession, and the lowest, at the shrub stage. In our 2021 work, it was indicated that the grass stage of regenerative succession displays the highest grass diversity (up to 32 species per plot) (Gorbunova, Sumina, 2021); the forest stage shows approximately the same number (up to 26 species). At the same time, minimal grass biodiversity is present at the pioneer and shrub stages. It appears that a greater grass species diversity is more beneficial for AMF due to a wider range of nutrient providers, which may eventually benefit both fungi and plants (Kiers et al., 2011). Similar results were shown in a succession study of a fungal community in a retreating glacier in the Cascade Range (Jumpponen et al., 2012). The authors note that when the mycorrhizal fungi diversity increases, the plant species diversity and the plant community primary production increase as well; this is also in line with earlier studies (van der Heijden et al., 1998). Our research indicates a strong negative correlation (–0.85) between the number of AMF species and the number of woody plant species. The number of tree species is highest at the shrub stage with the minimum number of AMF species. No correlation was found between herbaceous plant species and the number of AMF species. The latter is probably due to the large number of independent factors affecting vegetation and AMF (Gorbunova, Sumina, 2021).

According to earlier results, based on the microscopy study of samples (Gorbunova, Sumina, 2021), it was observed that the number of fungal propagules in soil and the diversity of mycorrhizal fungi species do not increase with succession; they even decrease at the shrub stage. This agrees in part with the data of the molecular genetic part of the study. It should be noted that the AMF count registered by microscopy is usually lower than that in molecular genetic studies (Kryukov et al., 2020).

M. Zobel and M. Öpik (2014) formulated the habitat hypothesis to distinguish the case where AMF and plant biodiversity are correlated but not in a direct cause-and-effect relationship, as opposed to the null hypothesis of no correlation (independence). For example, during primary succession, plants typically occupy the habitat before AMF and then act as a potential filter for AMF, i. e., AMF are “passengers”, as they follow the plants. However, limited distribution in a stable AMF community may result in the AMF community being a stronger determinant of which plants take root during secondary succession; in this case, the AMF community becomes the driver (Zobel, Öpik, 2014).

The biodiversity data from the two quarries in this study differ due to their different age. The Kalelovo quarry is fairly young with rather broken plots; the overgrowth process has been taking place for a shorter period; trees and shrubs are almost absent and young. The Kuzmolovo quarry has been overgrowing for about three decades; there are some bare plots, free of trees and shrubs, but tree regeneration is quite active (Gorbunova, Sumina, 2021). At the same time, different stages at the two quarries provide an opportunity to investigate how biodiversity changes during the development of the regenerative succession. During revegetation (shrub and forest stages), the competition between soil microbiota increases, and we see the results of ecological filtering of the fungal species introduced at the first stage, which changes the species composition, and AMF, more capable of symbiosis with grasses, tend to persist (Yurkov et al., 2024). R. irregularis tends to vanish during the grass stage; it is more common during an unusual pioneer stage and is eventually replaced by other AMF species. Still, this species is widespread, as other studies show, including our work on AMF biodiversity assessment in the North Caucasus (Yurkov et al., 2024). Compared to the AMF diversity in the Caucasus, it is lower in quarries, which is in line with the suggestion that AMF biodiversity is lower in sandy soils than in others (Ganugi et al., 2019).

Fairly many AMF species have been observed at the pioneer stage, as the ecological niches are vacant, and the species diversity is subject to random factors, mainly fungal introductions to plots (van der Heijden et al., 2015). The efficiency of AMF-plant symbiosis reaches maximum levels during the grass stage, while the number of AMF species is also at its maximum. During the shrub stage, grasses are replaced by shrubs and undergrowth associated with ectomycorrhizal fungi (aspen, birch, etc.); grasses are suppressed by the lack of light and the presence of woody plant roots in the soil, resulting in a dramatic decrease in AMF diversity. During the forest stage, grass species become slightly more abundant due to the accession of typical forest species, but competition for light and soil resources remains high, which gives a slight increase in AMF diversity (Neuenkamp et al., 2021).

The distribution of fungal biodiversity across taxonomic groups (Fig. 2) is influenced by both random factors (mainly at the pioneer stage) and competitive selection (at the later stages). During the pioneer stage, samples predominantly contain basidio- and ascomycetes, the spores of which spread much more easily than AMF spores, brought by wind, water, humans, and animals (Janowski, Leski, 2022). At the grass stage, the AMF proportion increases, as there are more plant species with which previously introduced AMF can enter into symbiosis. The maximum AMF percentage is attributed to their complementarity with grasses, high abundance of herbaceous plant species, and reduced competition among AMF. Soils become more fertile, and conditions become favorable for other fungi (Fig. 2), the proportion of which is maximum for this succession stage. In the shrub community, the AMF share is minimal, as well as the diversity and projective coverage of mycorrhizae-forming grasses; shrubs and undergrowth dominate here; they are proactive in forming a symbiosis with ectomycorrhizal fungi, which include many basidio- and ascomycetes (to a lesser extent). The picture is similar in plots with the forest stage, except that the trees are even more developed, which may influence the increased share of basidiomycetes; the shares of other groups are also significant, as the soils are more developed, and there is a lot of forest litter (Gorbunova, Sumina, 2021).

## Conclusion

Regenerative succession in quarries is represented by four consecutive overgrowth stages of the free substrate and woody vegetation regeneration: pioneer, grass, shrub, and forest stages. AMF biodiversity is high at the first stages of regenerative succession. The highest AMF diversity is observed at the grass stage of the succession development. This is primarily due to the significant number of herbaceous species with which AMF form better symbiotic relationships. The lowest AMF biodiversity at the shrub stage is caused by the fact that herbaceous plants (including grasses) do not grow well in the shrub shade which reduces AMF species and their abundance. As has been shown, the most widespread AMF at the pioneer stage is R. irregularis. Other AMF types then take advantage. The results show that the biodiversity and species composition of AMF can vary widely among stages of regenerative succession and are likely to depend primarily on grass biodiversity. Although there are different hypotheses as to whether it is the fungus or the plant that is in charge, our work shows that it is likely that plants determine which AMF will be associated with them, and not vice versa.

## Conflict of interest

The authors declare no conflict of interest.
